# Team behaviors as antecedents for team members’ work engagement in interdisciplinary health care teams

**DOI:** 10.3389/fpsyg.2023.1196154

**Published:** 2023-07-04

**Authors:** Sebastian Gerbeth, Regina H. Mulder

**Affiliations:** Faculty of Human Sciences, University of Regensburg, Regensburg, Germany

**Keywords:** work engagement, team learning behaviors, team emotions, work teams, structural equation model

## Abstract

**Introduction:**

Due to the increasing complexity and diversity of work tasks in teams, teams need team members who are dedicated and energetic, both characteristics attributed to team members’ work engagement. Especially in the domain of health care, high demands at work impact professionals’ work engagement. Despite teams being the main work unit in this domain, team research on antecedents of work engagement has been neglected. The present study examines the role of team behaviors such as reflection activities in the relationships between demands at work and team members’ work engagement. In doing so, the study aims to extend findings on team behaviors by considering cognitive and work-task related team behaviors as well as team behaviors that focus on emotional aspects.

**Methods:**

Data of 298 team members of 52 interdisciplinary teams of health and social care organizations which provide care and assistance were collected in this cross-sectional survey study. Relationships between team demands at work, team learning behaviors, dealing with emotions in the team and team members’ work engagement were estimated in a mediation model using structural equation modeling (SEM).

**Results:**

The results indicate that team members’ work engagement is positively related to team learning behaviors and dealing with emotions in the team. Cognitive team demands at work such as the complexity of work tasks, were found to relate positively to team members’ work engagement, while emotional team demands such as the amount of emotional labor at work had a negative relationship. Team learning behaviors and dealing with emotions in the team were found to mediate the relationship between team demands at work and team members’ work engagement.

**Discussion:**

Our results provide insights into the actual behavior of teams in the domain of health care, both on cognitive and emotional aspects, and the capability of team learning behaviors and dealing with emotions in the team to mediate the relationship between team demands at work and team members’ work engagement. The findings encourage future researchers and practitioners to address cognitive, emotional and motivational components in team research to provide a better understanding of team conditions, team behavior and team outcomes.

## 1. Introduction

Interdisciplinary teams have become an essential part of work in the last century and are needed to fulfill diverse tasks with high complexity (Van der Haar et al., 2008; Rosen et al., 2018). Particularly in the field of caring and assistance, teams in health and social care organizations often need to collaborate and cooperate with the patient, the patient’s family, physicians, psychologists, and other experts to ensure that the patient’s needs and goals are met. Teams are defined as a group of two or more individuals who interact socially to achieve common goals and perform content-related tasks while depending on each other (Kozlowski and Ilgen, 2006). Due to the increasing complexity and diversity of work tasks, teams need team members who are dedicated and energetic, both characteristics attributed to the team members’ work engagement. Work engagement is defined as a positive work related affective and cognitive state characterized by vigor, dedication, and absorption (Schaufeli et al., 2006). Engaged team members have a lot of energy and perseverance for their work tasks and are proud and enthusiastic about the team and their work in the team, which is especially important for the work carried out by teams in health care. Work engagement is related to various work outcomes such as performance, commitment, health and turnover intention (Halbesleben, 2010; Mazzetti et al., 2021; Neuber et al., 2022). Work engagement is a predictor of performance at individual level and team level in health and social care organizations (Tims et al., 2013). Engaged employees have higher performance (Halbesleben and Wheeler, 2008; Christian et al., 2011; Reina-Tamayo et al., 2018) and low levels of mental health such as anxiety and depression (Innstrand et al., 2012). Accordingly, work engagement is negatively related to turnover intention (De Simone et al., 2018; Wan et al., 2018).

Antecedents of work engagement are demands at work such as challenging and hindering demands. Challenging demands, which have the potential to contribute to performance and learning, have a positive effect on work engagement. Hindering demands, which create constraints that hinder the achievement of work goals, have a negative effect on work engagement (Cavanaugh et al., 2000; Mauno et al., 2007; Breevaart and Bakker, 2018; Riedl and Thomas, 2019; Uhlig et al., 2023). While at the individual level there is empirical evidence on how work engagement is influenced by antecedents and how it impacts various outcomes, the nested structure of organizations also highlights other levels, such as work teams. For example, employees in full-time residential homes for the disabled have to work in teams due to the full-time care of their patients. There is a lack of studies that consider especially variables at the team level as antecedents to team members’ work engagement, and that uses a multilevel perspective considering team members being influenced by the teamwork, their team leader and the team (Bakker and Demerouti, 2017). Therefore, the aim of this present study is to provide insights into the demands of teams and their influence on team members’ work engagement. Furthermore, the missing link, being the actual behavior of the team members and its impact on work engagement, needs further investigation. Insights into these relationships are lacking so far.

When teamwork occurs, team members are carrying out team behaviors that constitute team member interaction, such as discussing or reflecting on work. Team learning behaviors (TLBs) are defined as team activities team members are carrying out to effectively accomplish work tasks. Teams that share and exchange knowledge between their members, create common understandings and new knowledge, reach agreement by constructively combining and discussing opinions and reflect on their teamwork are recognized as teams carrying out a high amount of TLBs (Widmann and Mulder, 2020) and therefore, represent teams with high cognitive and work-task related team behaviors. TLBs lead to change and improvement in the team (Decuyper et al., 2010). In addition, TLBs were found to predict team performance (Leicher and Mulder, 2016) and team cognition such as shared mental models (Widmann and Mulder, 2020), and parts of TLBs such as team reflection were also found to affect work engagement (Matsuo, 2020; Gupta et al., 2022). Since the objective of this study is to provide insights into the role of team behaviors on team members’ work engagement, we focus on the actual interactions within the team instead of the outcomes of these interactions, such as team cognitions. In teams of health and social care organizations, team behaviors are observed to be not only cognitive and work-task related, but include emotional and social aspects (e.g., in patient care). Dealing with emotions in the team consists of activities such as commonly reflecting about occurring emotions in the team or expressing and influencing positive emotions such as gratitude. Dealing with emotions in the team represents team behaviors focused on emotional aspects and is so far neglected in team research.

Mathieu et al. (2019) highlighted the complexity and multilevel perspective of teamwork by recognizing team characteristics and demands, team behaviors and structural features as mediators of outcomes such as team effectiveness and performance. Employees carry out cognitive and work-task related team behaviors such as TLBs and team behaviors focused on emotional aspects such as the dealing with emotions in the team to cope with the variety of complex tasks and demands present in teamwork. The objective of the present study is to fill the knowledge gap on team behaviors as antecedents of individual’s outcomes by increasing insights into the role of team behaviors for the relationships between the demands that teams face in their work and team members’ work engagement in the domain of care and assistance. Therefore, the following research question will be answered:

*Do team learning behaviors and dealing with emotions in the team mediate the relationships between team demands at work and team members' work engagement?*

To answer this research question, we formulated three sub-questions:

*To what extend do teams that provide care and assistance to the elderly, youth, physically and/or mentally disabled engage in team learning behaviors and dealing with emotions in the team?*

*Do team learning behaviors and dealing with emotions in the team predict team members' work engagement?*

*Do team demands at work predict team learning behaviors and dealing with emotions in the team?*

## 2. Theoretical background

### 2.1. Work engagement

The concept of work engagement was first pioneered by Kahn (1990) describing employees’ work engagement as the employment and expression of personal energies to emotional, cognitive and physical labor. Engaged employees become physically involved in tasks, emotionally connected to other employees relevant for their work and cognitively vigilant, while disengaged employees withdraw and show passive behavior that is characterized by physical absence, a lack of emotional connections and cognitive inattention. Due to the behavior of disengaged employees, work engagement is also considered the opposite of Burnout (Maslach and Leiter, 2008), but Schaufeli et al. (2002) argue that work engagement is distinct from burnout, which is characterized by vigor, dedication, and absorption. Vigor is defined as an employee’s energy, mental resilience while working, and willingness to invest effort. Dedication refers to the degree of enthusiasm, inspiration, pride, and appreciation for an employee’s own work, and absorption describes a state of being completely focused and fully involved in one’s work.

Based on Vroom’s (1964) Expectancy Theory, team members’ motivation, which can foster their work engagement, depends on the demands at work and the belief that the team member will successfully cope with them (expectancy), associated with the belief that coping with the demand will lead to an outcome (instrumentality) that is valued or attractive (valence). Accordingly, work engagement can be influenced by behaviors of the team members themselves and the processes that occur in teams through the interactions of team members. These interactions between team members can be related to cognition (e.g., in team learning behaviors) and can be related to emotions (e.g., in dealing with emotions in the team). This is consistent with research that indicates that inputs are transformed into outcomes such as work engagement through cognitive, verbal, emotional, and behavioral processes (Marks et al., 2001). In addition, the degree of interaction is central as fewer opportunities for interaction lead to fewer experience of vigor, dedication, and absorption within the work (Costa et al., 2012).

### 2.2. Team learning behaviors

According to Vygotsky’s (1978) Sociocultural Theory, learning, and especially learning in the workplace occurs in social interactions. Thereby, cognitive and social processes influence individual learning and development embedded in teams and organizations (Van den Bossche et al., 2006). Team learning is defined as interplay of complex and dynamic team level processes that lead to change or improvement for teams and their members (Decuyper et al., 2010) and can directly influence team outcomes such as performance and shared mental models (Leicher and Mulder, 2016; Widmann and Mulder, 2020). These processes consist of TLBs referring to team activities team members are carrying out such as sharing, discussing and developing knowledge, ideas and structures and obtaining feedback and reflecting (Edmondson, 1999; Lehmann-Willenbrock, 2017).

There are three basic team learning behaviors (knowledge sharing, co-construction and constructive conflict) that are crucial for the team’s function as “they describe what happens when teams learn” (Decuyper et al., 2010, p. 117). Wiese et al. (2022) argue that knowledge sharing is different from co-construction and constructive conflict because knowledge sharing is an important prerequisite for co-construction and constructive conflict, but is not sufficient for a team to learn. *Knowledge Sharing* refers to the exchange of knowledge and structures between team members and can help teams to reach a common knowledge level (Widmann and Mulder, 2020). *Co-construction* is defined as team activities that lead to the creation of new knowledge, structures or common meanings in the team by refining, building on or modifying knowledge, experiences and information (Van den Bossche et al., 2006). *Constructive conflict* describes the process of reaching agreement in the production of new knowledge, recognizing that different team members may not always coincide and therefore some form of team agreement must be reached (Decuyper et al., 2010; Raes et al., 2015). Constructive conflict addresses the handling of different opinions by open communication, negotiation and verification in form of directly commenting or asking critical questions (Van den Bossche et al., 2011). *Team reflection* is defined as reflection and discussion activities on current teamwork, goals, structures and how to adapt as a team for the achievement of future work goals (Decuyper et al., 2010). Team reflection is a facilitating team learning behavior providing context for the basic team learning behaviors (Raes et al., 2015).

Organizations, teams and team leaders affect team members’ work engagement by creating job resources (for example support, autonomy or group cohesion) that could be used to deal with work tasks (Bakker, 2017; Tummers and Bakker, 2021). When carrying out TLBs team members are interacting, reflecting, developing and working together which could foster social relatedness (e.g., promoting dialogue and exchange), the feeling of competence (e.g., promoting the creation of common vision, optimizing team structure and work processes, and the fulfillment of work tasks) and the feeling of autonomy (e.g., creating individual learning opportunities, encouraging to contribute own opinions, experiences, knowledge and ideas). Therefore, TLBs can be considered as an underlying resource mechanism that fosters the basic needs for autonomy, relatedness and the feeling of competence formulated in the Self-Determination Theory (Deci and Ryan, 2000), which postulates that motivation can be increased by satisfying the basic needs. In turn, motivated employees have higher levels of work engagement (Shkoler and Kimura, 2020). Furthermore, satisfaction of basic needs itself yields positive work outcomes such as work engagement, well-being and enhanced work performance (Deci et al., 2017).

Furthermore, referring to Flow Theory (Csikszentmihalyi, 1990) individuals can experience flow during activities, that are characterized by a deep involvement in a task while experiencing feelings of energy, focus and success in the process of task completion. Studies found positive correlations between experiencing flow and outcomes such as job satisfaction, intrinsic motivation, and vigor (Csikszentmihalyi and LeFevre, 1989; Demerouti et al., 2012). There has been an increasing interest in flow in work teams, as teams (through their complex tasks, common goals, and interdependencies) engage in team activities that fulfill the preconditions for flow experiences (Walker, 2010; van den Hout et al., 2018, 2019). We argue that TLBs are potential team activities that could lead to flow experiences in teams or within team members as TLBs are goal-directed, occur in cognitive demanding tasks and are based on mutual commitment, open communication and trust (Decuyper et al., 2010). This is in line with the reciprocal relationships between TLBs and positive emotions such as pleasure, confidence, solidarity, and contentment during teamwork (Watzek et al., 2022).

Therefore, we formulate:

*Hypothesis 1*. Team learning behaviors are positively related to work engagement.

### 2.3. Dealing with emotions in the team

In addition to cognitive processes in the team, processes in relation to emotions in the team can influence work engagement. At the individual level emotional competence and emotional intelligence that consists of the perception of own or others’ emotions, the expression, and the management of emotions (Stamouli, 2014; Mayer et al., 2016), were found to influence employee’s work engagement (Gong et al., 2020; Tesi, 2021). Mindeguia et al. (2021) found team emotional intelligence to have a positive effect on passion and group cohesion, that as job resource is an antecedent of work engagement (Costa et al., 2014; Tesi, 2021). The concept of team emotional intelligence is examined by differences in “the ability of a group to generate a shared set of norms that manage the emotional process in a way that builds trust, group identity and group efficacy” (Druskat and Wolff, 2001, p. 138). Existing research highlights that emotions have been recognized as crucial factors in teams and organizational dynamics (Kelly and Barsade, 2001; Menges and Kilduff, 2015). There is research on the role of emotions in teams (e.g., Cahour, 2013; Watzek and Mulder, 2019), but studies that investigate what teams actually do when team members are confronted with emotions during teamwork are missing.

Team processes related to emotions in teams are characterized by behaviors of team members to commonly perceive emotions, express and regulate emotions occurring during teamwork. Thereby, team members themselves shape their collective emotional experiences through their interactions and behavior, leading to the emergence of shared norms and expectations within the team (Wolff et al., 2006). Therefore, *dealing with emotions in the team* is defined as team activities, shared by at least two team members, focused on emotions that arise in the team. *Dealing with emotions in the team* consists of team activities such as discussing, reflecting, or exchanging about the emotions in the team, for instance to understand and recognize present emotions and to cope with encountered emotions in the team. In addition, team activities of expressing and reacting to emotions, for instance to be sensitive to the emotions of the team members, to express positive and negative emotions and to actively influence emotions.

Bakker (2022) posits that the social-psychological construct of emotional contagion is as an explanatory approach to the emergence of work engagement in teams. Based on the concept of emotional contagion that refers to processes whereby emotions are transferred among team members (Barsade, 2002) it is argued that dealing with emotions in the team can influence team members’ affects and behaviors. Additionally, recognizing work engagement as a positive affect (high levels of activation and pleasure; Bakker and Oerlemans, 2011) which can be observed by other team members could in accordance with the Emotion As Social Information Theory (Van Kleef, 2009) lead to further work engagement of other team members. Following the different theoretical foundations, and in line with results indicating that emotions in teams increases performance (Watzek et al., 2022) the expectation is that team members’ work engagement is increased by dealing with emotions in the team, which leads to the second hypothesis:

*Hypothesis 2*. Dealing with emotions in the team is positively related to work engagement.

### 2.4. Demands at work

Team members face a variety of job demands that determine their teamwork and the work of each team member separately. Demands at work can be classified as either quantitative or qualitative in nature. Quantitative demands refer to the *amount of work* that needs to be accomplished within a certain amount of time and the *work pace* that refers to the speed and urgency of tasks to be fulfilled (Kristensen et al., 2004). Qualitative demands refer to the content of work such as cognitive demands and emotional demands. *Cognitive demands* refer to the complexity of tasks and the amount of problem-solving and decision-making required for accomplishing tasks, whereas *emotional demands* arise from interactions with clients and colleagues, which can be emotionally stressful (Crawford et al., 2010; Bakker and Demerouti, 2017).

Crawford et al. (2010) identified inconsistencies in the research on the relationships between demands at work and work engagement, that could be explained by the Transactional Theory of Stress (Lazarus and Folkman, 1984). This theory posits that individuals appraise stressful situations as either threatening or promoting for mastery and growth. The challenge and hindrance framework of Cavanaugh et al. (2000) supports this reasoning by differentiating between challenging demands, that are appraised as potential to contribute to achievement and learning by creating positive feelings of fulfillment, and hindering demands, that create constraints that hinder work goal achievement. Combined with the aforementioned Expectancy Theory (Vroom, 1964) challenging demands are positively related to work engagement. In contrary, hindering demands are negatively related to work engagement. Emotional demands in nursing and care, for example, require a high level of emotional labor (e.g., calming down an angry patient) that may be overwhelming and exhausting, and as a result may threaten a team member’s motivation to continue working with the patient.

In addition, in practice work pace and cognitive demands were found to be positively related to work engagement, while the amount of work and emotional demands are negatively related to work engagement (Crawford et al., 2010; Breevaart and Bakker, 2018; Riedl and Thomas, 2019; Uhlig et al., 2023). Accordingly, based on this argumentation the amount of challenging and hindering demands influence TLBs and dealing with emotions in the team. While the amount of work in the team and tasks to be done may decrease sharing or collaborative interaction due to splitting of work tasks, we postulate that cognitive demands, which describe the complexity of the work tasks to be done, require increased collaboration and cooperation and lead to more discussion to reach agreement, thereby increasing team activities. Therefore, we assume that demands at work influence TLBs, dealing with emotions in the team and team members’ work engagement.

*Hypothesis 3a.* The amount of work is negatively related to TLBs, dealing with emotions in the team and team members’ work engagement.

*Hypothesis 3b.* Work pace is positively related to TLBs, dealing with emotions in the team and team members’ work engagement.

*Hypothesis 3c.* Cognitive demands at work are positively related to TLBs, dealing with emotions in the team and team members’ work engagement.

*Hypothesis 3d.* Emotional demands at work are negatively related to TLBs, dealing with emotions in the team and team members’ work engagement.

*Hypothesis 4.* TLBs and dealing with emotions in the team mediate the relationship between demands at work and team members’ work engagement.

**Figure 1:**
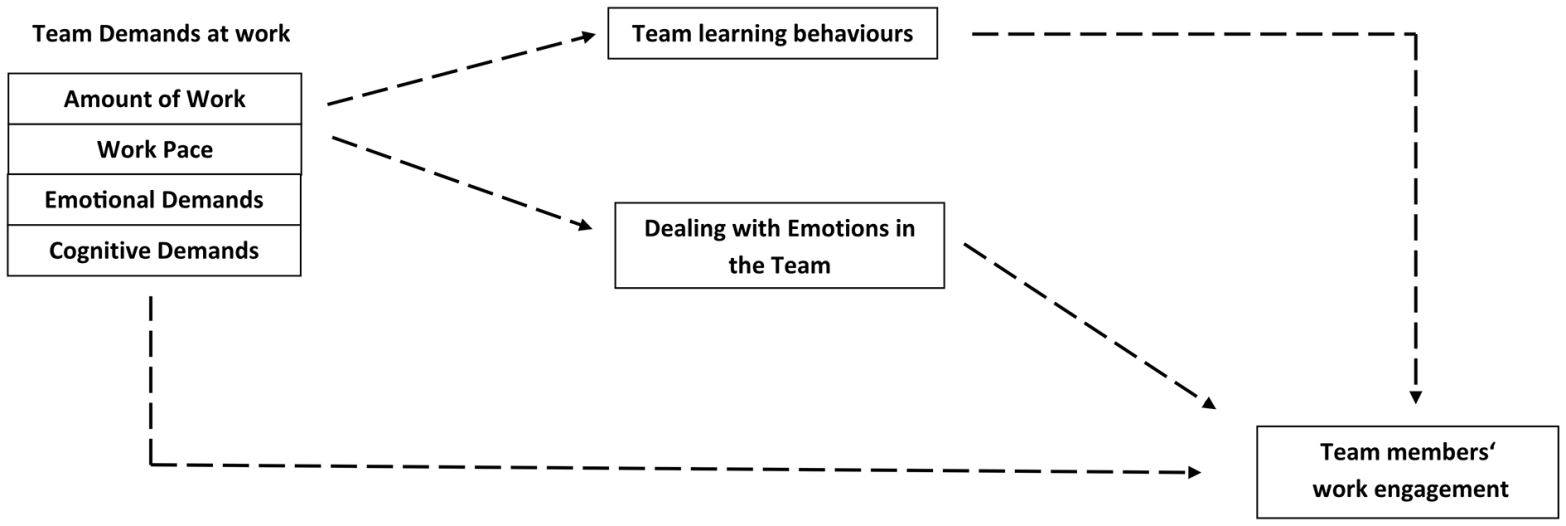
Research model.

To answer our research question Figure 1 presents our research model.

## 3. Materials and methods

### 3.1. Study design and data collection

A cross-sectional survey was carried out with a questionnaire in an online as well as paper version. Teams from eight different organizations, that provide care and assistance to the elderly, youth, or physically and/or mentally disabled, were invited to participate. Data were collected from teams whose work tasks are delivering care (e.g., in full-time residential homes), nursing and assistance of people (e.g., treatment according to medical prescription for disabled). Furthermore, teams were selected that met the previously mentioned definition: (1) the team and their members have a common work goal; (2) team members are interdependent in fulfilling their work tasks for the goal; (3) the team consists of more than 3 team members; (4) team members consider themselves to be permanent members of the team. Informal consent was obtained prior of the study by all participants. Participation in the study was voluntary, and all participants were fully informed about the study prior to the data collection. Anonymity of participants, teams and organizations was maintained at all times by pseudonymizing the teams and organizations. No personal information (e.g., names, email) was gathered from the team members. Ethical approval was granted by the ethics committee of the university of Regensburg (no. 22-3077-101).

### 3.2. Sample

Team members (*N* = 298) from 52 different teams participated in the study. 78.8% of the participants were female (1 missing) and the average age was M (SD) = 40.23 (12.41; 9 missing). 42.1% of the team members were nurses, 28.3% were social or childcare workers, 2.4% were psychologists, 1% were team leaders and 23.1% were assistants. The average amount of work experience was M (SD) = 13.79 (11.26) years. For 23.5% the last job change was in the last 2 years, while 17% had their last job turnover over 10 years ago.

Teams had an average of *M* (SD) = 12.49 (6.27) team members and ranged from 4 to 28 team members. Most of the team members (59.9%) entered their team more than 2 years ago, while 23.9% did so in the last year. 5.9% joined their team in the last 3 months prior to the data collection (these were consistent with the participants who changed jobs in the last 3 months). Furthermore, in relation to team stability, it was found that 52.2% of the teams had the last gain of a team member over 3 months ago, and for 52,7% of the teams the last loss. This is consistent with the high employee turnover rate in the organizations in the field of caring. In addition, 71.9% of the participants of the study reported that they joined their team over 1 year ago. In the data there were no *ad hoc* or newly formed teams that were not able to report adequate data for our study.

### 3.3. Instrument

The questionnaire contained the following variables: *Team members’ work engagement* was measured with the short version of the Utrecht Work Engagement Scale (UWES-9; Schaufeli and Bakker, 2004) containing nine items on the extent to which employees identify with, are proud of, and enthusiastic about their work, and have a lot of energy and perseverance for their work tasks. With a 7-point Likert-type response format (1 = never to 7 = daily) the frequency of experiencing the three facets of vigor, dedication and absorption of work engagement was measured. An example item is: “*At my work, I feel bursting with energy*.” Cronbach’s α was 0.93.

The Copenhagen Psychosocial Questionnaire (COPSOQ II; Pejtersen et al., 2010; COPSOQ III; Lincke et al., 2021) was used to measure *team demands at work*. We adapted items of five scales of the German version of the COPSOQ III including the amount of work (4 items), work pace (3 items), cognitive demands (4 items), emotional demands (3 items). A 5-point Likert-type response format (1 = never to 5 = always) was used with a reference shift to the team level as for example “*does your team get behind with the work*” (amount of work). The Cronbach α’s ranged from 0.65 to 0.87.

*Team learning behaviors* were measured with items covering *knowledge sharing*, *co-construction, constructive conflict,* and *team reflection*. *Knowledge sharing* was measured with eight items of Neumann (2017) with a Cronbach’s α = 0.86. Co-Construction and constructive conflict were measured with ten items each (Widmann et al., submitted) with Cronbach’s α ranging from 0.87 to 0.91. *Team reflection* was measured with eight items of Van Dick and West (2005; Cronbach α = 0.87). Example items are: “*we pass on task-relevant know-how in the team*” (knowledge sharing), “*we draw conclusions from the ideas discussed in the team*” (co-construction), “*we try to address disagreements in the team directly*” (constructive-conflict) and “*we regularly discuss whether the team is working together effectively*” (team reflection). The Likert-type response format ranged from 1 = “never” to 5 = “always.”

For measuring *dealing with emotions in the team* we developed a scale with 32 items (Gerbeth et al., in preparation) that measure team activities such as discussing, reflecting, or exchanging about emotions (e.g.: *“we ask each other about the reasons for our current emotional state”*, “*we reflect on emotional events that have engaged us as a team”*) and expressions and reactions to emotions (e.g.: *“in our team we express our gratitude to each other for good work”*, “*in our team, we respond sensitively to the emotions of team members*”). For assessing the frequency of engagement of the team in these activities a 5-point Likert-type response format mode ranging from 1 = “never” to 5 = “always” was used. Three items were removed due to poor quality and reliability. An exploratory factor analysis revealed a single-factor solution accounting for 42.06% of the variance. Cronbach’s α was 0.95.

Furthermore, the control variables team size, team stability, gender, age and work experience (in years) were included in the questionnaire.

### 3.4. Analyses

Descriptive statistics and correlation analysis were carried out using IBM’s SPSS Statistics 29 software. Because the data of team members in work teams are nested, within-group agreement using the multiple-item estimator (r_wg(j)_) and the intraclass correlation coefficient (ICC) for constructs at team level was calculated. The r_wg(j)_ and ICC values for TLBs, dealing with emotions in the team and team demands at work are presented. For TLBs (r_wg(j)_ = 0.87–0.94), dealing with emotions in the team (r_wg(j)_ = 0.96) and team demands at work (r_wg(j)_ = 0.81–0.96) the r_wg(j)_ values exceeded the proposed cut of value for aggregation of 0.70 (LeBreton and Senter, 2008). For ICC(1) the values of TLBs (ICC(1) = 0.15–0.26), dealing with emotions in the team (ICC(1) = 0.20), and team demands at work (ICC(1) = 0.21–0.44) exceeded the cut-off value of 0.12 (Bliese, 2000) while for ICC(2) the values varied from 0.51 to 0.82.

Structural equation modelling (SEM) was performed using MPLUS 8.2 with robust maximum likelihood estimation and the “type = complex” setting for nested data structure to adjust the standard errors of the regression coefficients (see Muthen and Satorra, 1995). The items were used as indicators of latent variables. For model estimation due to parsimony, item parceling for TLBs and dealing with emotions in the team was conducted by averaging scores of content related and substantially correlated items (Little et al., 2002). In the initial model team size, work experience and team membership were controlled for, but as there were no meaningful significant effects these variables were excluded in the following analyses due to parsimony. Because the *χ*^2^-test is sensitive for moderate to large sample sizes (Chen, 2007), the comparative fix index (CFI), the root mean square error of approximation (RMSEA) and the standardized root mean squared residual (SRMR) are reported next to the *χ*^2^ value for evaluating model fit of the structural equation models tested. We follow the recommendation of Hair (2014) that RMSEA values smaller than 0.08, SRMR values smaller than 0.10 and CFI values higher than 0.90 are satisfactory model fit and RMSEA values smaller than 0.06, SRMR values smaller than 0.08 and CFI values higher than 0.95 are good model fit. Respondents with missing data were removed prior to SEM analysis.

## 4. Results

### 4.1. Descriptive statistics and correlations

In Table 1 the means, standard deviations, Cronbach’s alphas and zero-order correlations of all variables are listed. Team members reported high levels of work engagement. Based on the dimension scores according to the UWES norm (Schaufeli and Bakker, 2004) vigor is average (*M* = 4.75, SD = 1.35), dedication is high (*M* = 5.23, SD = 1.40), and absorption is high (*M* = 4.89, SD = 1.49). Furthermore, the mean scores indicate that teams and team members strongly engage in knowledge sharing, co-construction, constructive conflict and dealing with emotions in the team. In accordance with our assumption that these teams are particularly engaged in complex cognitive work tasks, team members reported high cognitive demands at work. Female respondents had higher levels of work engagement than males (*T*-test (*df*) = 2.71 (286), *p* < 0.01). There were no relevant significant relationships with age. Work experience (in years) relates negatively to work engagement, TLBs and dealing with emotions in the team, while there was a positive relationship with demands at work. Team members who joined their team recently reported higher work engagement while team members who had worked in their team for a few years reported higher amounts of work pace and less knowledge sharing. For team stability no relationships were found. For team size we found correlations with work engagement, cognitive demands and work pace. Correlation coefficients for team size, work experience and joining team are presented in Table 1.

**Table 1:** Descriptive statistics, internal consistency and zero-order correlations.

Variables	*M*	SD	1	2	3	4	5	6	7	8	9	10	11	12	13
1. Work engagement	4.96	1.31	*0.93*												
2. Demands at work	3.28	0.52	0.08	*0.87*											
3. Quantitative demands	2.95	0.50	−0.05	0.66^**^	*0.75*										
4. Work pace	3.05	0.85	0.07	0.74^**^	0.34^**^	*0.84*									
5. Cognitive demands	3.79	0.74	0.22^**^	0.83^**^	0.34^**^	0.49^**^	*0.81*								
6. Emotional demands	3.26	0.71	−0.05	0.76^**^	0.43^**^	0.34^**^	0.56^**^	*0.65*							
7. Knowledge sharing	4.05	0.65	0.29^**^	0.14^*^	−0.03	0.11	0.28^**^	−0.01	*0.86*						
8. Co-construction	3.73	0.69	0.30^**^	0.15^**^	−0.06	0.15^*^	0.30^**^	−0.02	0.86^**^	*0.91*					
9. Constructive conflict	3.65	0.65	0.27^**^	0.07	−0.13^*^	0.09	0.23^**^	−0.06	0.81^**^	0.86^**^	*0.87*				
10. Team reflexion	3.28	0.73	0.29^**^	0.25^**^	0.01	0.20^**^	0.35^**^	0.12^*^	0.65^**^	0.78^**^	0.67^**^	*0.87*			
11. Dealing with emotions in the team	3.52	0.61	0.33^**^	0.19^**^	−0.01	0.16^**^	0.29^**^	0.08	0.75^**^	0.78^**^	0.76^**^	0.67^**^	*0.95*		
12. Work experience^1^	13.79	11.26	−0.15^**^	0.15^*^	0.05	0.15^*^	0.10	0.13^*^	−0.19^**^	−0.19^**^	−0.12	−0.15^*^	−0.15^*^		
13. Joining team^2^	3.78	1.45	−0.26^**^	0.13^*^	0.10	0.17^**^	0.01	0.11	−0.12^*^	−0.11	−0.08	−0.01	−0.05	0.41^**^	
14. Team size	12.49	6.27	0.16^**^	0.13^*^	0.00	0.13^*^	0.18^*^	0.05	0.03	0.02	0.00	0.04	−0.02	0.01	−0.20^**^

*N* = 289 (Teams > 33% or min. Three team members), Cronbach α (internal consistency) italic on the diagonal. ^1^*N* = 281 and ^2^*N* = 274 due to missing data. ^**^ = *p* < 0.01, ^*^ = *p* < 0.05.

The correlations indicate positive relationships between work engagement and the TLBs knowledge sharing (*r* = 0.29, *p* < 0.01), co-construction (*r* = 0.30, *p* < 0.01), constructive conflict (*r* = 0.27, *p* < 0.01), team reflection (*r* = 0.29, *p* < 0.01) as well as with dealing with emotions in the team (*r* = 0.33, *p* < 0.01). These correlations are in accordance with the research model. Positive relationships were found between cognitive demands and TLBs (*r* = 0.23 to 0.35, *p* < 0.01) as well as dealing with emotions in the team (*r* = 0.29, *p* < 0.01). There were positive correlations between work pace and co-construction, team reflection and dealing with emotions in the team (*r* = 0.15 to 0.20, *p* < 0.05). The correlation analysis (Table 1) found high correlations among variables relating to team demands at work variables and between TLBs and dealing with emotions in the team. For reasons of potential multi-collinearity all predictor variables of team members’ work engagement were centered, and the variance inflation factor (VIF) was checked. Demands at work variables did not exceed the VIF value of 2.5 (Johnston et al., 2018). Because the VIF values for TLBs and dealing with emotions in the team exceeded 2.5, separate models for TLBs and dealing with emotions in the team were tested to avoid problems with multicollinearity.

### 4.2. SEM

**Figure 2:**
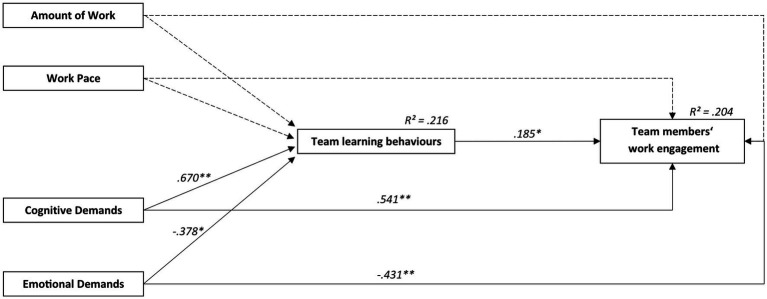
Relationships between team demands at work, team learning behaviors and team members’ work engagement analyzed with structural equation modelling. Model-fit: *X*^2^ = 472.387, *df* = 260, CFI = 0.951, RMSEA [CI] = 0.053[0.045–0.061], SRMR = 0.062. ^**^p < 0.01, ^*^p < 0.05.

The model for TLBs (see Figure 2) achieved a good model fit (*N* = 298 team members, *n* = 51 teams; *χ*^2^ = 472.387, *df* = 260, *p* < 0.001; CFI = 0.951; RMSEA [CI] = 0.053 [0.045–0.061]; SRMR = 0.062). TLBs were related to team members’ work engagement (*β* = 0.19, *p* < 0.01). The results support H1. Additionally, team members’ work engagement was found to be positively related to cognitive demands (*β* = 0.54, *p* < 0.01) and negatively related to emotional demands (*β* = −0.43, *p* < 0.01). In total, *R*^2^ = 0.204 of the variance of team members’ work engagement was explained by the model. The results indicate positive relationships between cognitive demands and TLBs (*β* = 0.67, *p* < 0.01) and negative ones between emotional demands and TLBs (*β* = −0.43, *p* < 0.01). Therefore, H3c and H3d was supported. No relationships between the amount of work, work pace and team members’ work engagement were found. So, there was no support for H3a and H3b. TLBs partially mediate the relationships of team members’ work engagement with cognitive demands (indirect effect *β* = 0.12, *p* < 0.01) and with emotional demands (indirect effect *β* = −0.07, *p* < 0.05). These findings in part support hypothesis H4.

**Figure 3:**
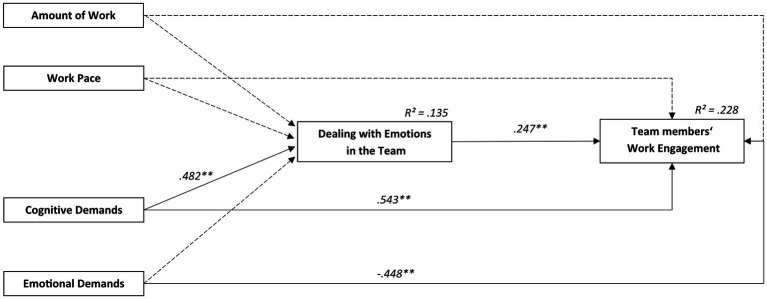
Relationships between team demands at work, dealing with emotions in the team and team members’ work engagement analyzed with structural equation modelling. Model-fit: *X*^2^ = 353.249, *df* = 194, CFI = 0.953, RMSEA [CI] = 0.053[0.044–0.062], SRMR = 0.054. ^**^p < 0.01, ^*^p < 0.05.

The model for dealing with emotions in the team (see Figure 3) revealed a good fit (*N* = 298 team members, *n* = 51 teams; *χ*^2^ = 353.249, *df* = 194, *p* < 0.001; CFI = 0.953; RMSEA [CI] = 0.053 [0.044–0.062]; SRMR = 0.054). The results indicate a positive relationship between dealing with emotions in the team and team members’ work engagement (*β* = 0.25, *p* < 0.01) which supports H2. Furthermore, team members’ work engagement was related positively to cognitive demands (*β* = 0.54, *p* < 0.01) and negatively to emotional demands (*β* = −0.45, *p* < 0.01). In total, *R*^2^ = 0.228 of the variance of team members’ work engagement was explained by the model. The results indicate a positive relationship between cognitive demands and dealing with emotions in the team (*β* = 0.48, *p* < 0.01) and team demands at work explained *R*^2^ = 0.135 variance of dealing with emotions in the team. Thus, the prediction of hypothesis H3c that cognitive demands at work positively relates to dealing with emotions in the team and team members’ work engagement was supported. H3a, H3b and H3d were not supported as no relationships were found with the amount of work, work pace and emotional demands. Furthermore H4, was partially supported by the indirect effect of team demands at work on team members’ work engagement that was mediated by dealing with emotions in the team (*β* = 0.12, *p* < 0.01).

## 5. Discussion

### 5.1. Antecedents of work engagement

Researchers recognized the complexity and multilevel perspective of team behaviors including cognitive, work-task related, emotional and social aspects (Bell, 2007; Mathieu et al., 2019). The present study increases insights into team antecedents of team members’ work engagement but also addresses team behaviors in work teams of health and social care organizations and investigates their role for the relationships between team demands at work and team members’ work engagement. Furthermore, insights into the demands at work of teams responsible for providing care and assistance to the elderly, youth, or physically and/or mentally disabled were provided.

The present study investigated team members’ work engagement based on the three facets vigor, dedication, and absorption. Results indicate that team members that provide care and assistance have high dedication and absorption to their work and intermediate vigor, which are slightly higher than findings before and during the COVID-19 pandemic (Riedl and Thomas, 2019; Bartsch et al., 2021). Team members reported that team demands at work such as the amount or pace of work are still high, but our results indicate that these are slightly lower to other studies (Riedl and Thomas, 2019). The findings that cognitive and emotional demands were also reported as high may be due to the fact that teams in these domains deal with many complex cognitive decisions and, therefore, need to take many aspects into account. Since all this happens in the context of social interactions with patients and their relatives, the work has the potential to be emotionally stressful. Therefore, it is particularly important to have team members who devote themselves to these diverse and complex tasks with high concentration, dedication and energy and who do not lose their capacity to work due to excessive emotional burdens.

Teams involved in care and assistance share knowledge, create new knowledge, structures, and plans through co-construction, achieve agreement through constructive conflict and are also characterized by a high level of reflective activities. The team members reported high knowledge sharing, co-construction, and constructive conflict but moderate team reflection activities. One explanation for this might be the working conditions of the teams, as many of them work in shifts which can hinder joint reflection activities. In line with Self-Determination Theory (Deci and Ryan, 2000) and Flow Theory (Csikszentmihalyi, 1990) teams that strongly engage in TLBs show higher work engagement. These results are consistent with studies in other domains and other types of teams investigating parts of TLBs and work engagement (Matsuo, 2020; Gupta et al., 2022; Peeters et al., 2022). Furthermore, our findings indicate that all TLBs (i.e., knowledge sharing, co-construction, constructive conflict, and team reflection) are carried out with similar frequency and positively related to work engagement. One explanation for that finding might be that TLBs are highly interrelated which is in line with Decuyper et al.’s (2010) team learning model and empirical studies (Widmann and Mulder, 2020). In addition, the findings that work experience is negatively related to TLBs may lead to the assumption that team members with long years of work experience fall into routines that result in less knowledge sharing, have less interest in contributing to developing new knowledge or achieving agreement, and reflect less. This also applies to dealing with emotions in the team, which suggests that team members with a lot of work experience participate less in team interactions where emotions are discussed.

The present study successfully measured dealing with emotions in the team and provides new insights into what teams actually do in relation to emotions. Thereby, our study makes a significant contribution to closing the gap that dealing with emotions in teams is detached from individual emotional competence as described by Elfenbein (2006). Our findings provide insights that extend the prior work of Druskat and Wolff (2001) and Aritzeta et al. (2020) on ‘team emotional intelligence’ while this present study does also take into account team-level emergence and focused on actual behaviors. Teams that discuss and exchange about emotions within the team and express emotions provide individual team members many opportunities for observing and reacting to emotions such as work-related pride and joy or being infected by these emotions. In line with emotion contagion (Barsade, 2002), Emotion As Social Information Theory (Van Kleef, 2009) and empirical studies (Holtz et al., 2020; Mindeguia et al., 2021), our findings support the assumption that team members’ work engagement is increased by team activities whose goal is to express, respond to, or discuss or share emotion within the team.

The current evidence also suggests that dealing with emotions in the team is strongly related to TLBs for teams that provide care and assistance, which was surprising at first. TLBs and emotional competence at individual level and at team level are moderately positively related (Gerbeth et al., 2022), suggesting that dealing with emotions in the team, which measures actual observable activities as perceiving, discussing, expressing and reacting to emotions, is also only moderately positively related to TLBs. In the domain of caring and assistance to elderly, youth, physically and/or mentally disabled, work tasks of teams are often linked to emotional aspects (e.g., decisions concerning a patient and his family). TLBs that are work task related could overlap with dealing with emotions in the team for work tasks that are directed to the handling of emotions occurring for example in patient interaction. Nevertheless, our results indicate differences in TLBs and dealing with emotions in the team, as dealing with emotions in the team explained more variance and had a stronger effect on team members’ work engagement than TLBs. These results indicate that emotional aspects are crucial in teamwork and that teams should not only focus on cognitive processes, but also recognize the team itself as a social unit and give space to dealing with emotions in the team.

Due to the aforementioned similarities and differences between TLBs and dealing with emotions in the team, it may be suggested that cognitive and emotional aspects are closely related in actual behavior in teams and that these are also related to motivational aspects. The results of this study lead us to strive for team research that extends previous research models by including cognitive, emotional and motivational components, which contributes to the call to consider cognitive, motivational and emotional factors as essential for learning outcomes within teams, such as team performance (Bell, 2007; Mathieu et al., 2019).

### 5.2. Limitations and future research

This study comes with limitations that should be addressed in future research. First, the cross-sectional design of our study was necessary to identify differences between cognitive work-task related team behaviors and dealing with emotions in the team. Nevertheless, determining changes and team dynamics was not possible. In future studies, longitudinal designs could fill this gap and help to validate the identified relationships over time. Second, we collected data from health and social care organizations and teams in the field of care and assistance to elderly, youth, people with physical and/or mental disabilities. Emotional labor is considered an important part of the teams’ field of activity and was decisive in determining the sample. In the context of the study, however, this circumstance could have led to a bias in the demands at work, since cognitive demands (e.g., decisions) and the amount of work can reciprocally influence the emotional demands. Furthermore, we recognize that the classification of a demand as a challenge or as a hindrance demand relies on the appraisal of the team member. This is not captured in the instrument that was used in this study. Replication studies with teams from other domains with less emotional labor in the work tasks could help to cross-validate the findings. Third, dealing with emotions in the team turned out to be good in covering activities focused on discussing, reflecting about emotions and expressing and reacting to emotions, however this variable needs further validation, also in different domains. Interestingly, although dealing with emotions in the team predicted team members’ work engagement, only cognitive demands were found to influence dealing with emotions in the team, and not emotional demands. Even tough teamwork is perceived as emotionally demanding it seems it might only have little influence on dealing with emotions in the team. Team members with high emotional competence have a better understanding of the harmful effects of emotionally demanding situations on their work engagement (Costa et al., 2014; Mayer et al., 2016). Therefore, future studies are needed to investigate relationships between dealing with emotions in the team and team members’ emotional competence. Fourth, due to multicollinearity (VIF values), it was not possible to test a model with both TLBs and dealing with emotions in the team at once. Further studies with larger datasets should address TLBs and dealing with emotions in the team and their effects in more detail to provide further insights into the relationships between cognitive behaviors and the teams’ dealing with emotions in the team. Therefore, replication studies in different domains and teams using multigroup analysis would be beneficial to cross-validate the findings. In addition, additional job characteristics such as full/part time, virtual versus face-to-face and other contextual variables at individual level (e.g., burnout, performance), at team level (e.g., psychological safety, team cohesiveness), and at organizational level (e.g., organizational commitment, organizational climate) may also be related to team members’ work engagement, TLBs and dealing with emotions in the team and should be examined in future studies. Furthermore, we suggest including multilevel analysis to investigate cross-level relationships that take into account the multilevel nature of team members nested in teams nested in organizations.

### 5.3. Practical implications

For teams and their members our results indicate that in the domain of care and assistance it is for fostering work engagement necessary to not only focus on the individual, but also on the team. Team members need to be aware that work engagement can be fostered by TLBs and dealing with emotions in the team. Moreover, TLBs and dealing with emotions in the team are important because they mediate the effects of team demands at work on work engagement. Teams that frequently carry out team activities of TLBs and dealing with emotions in the team reduce the effects of hindering demands on work engagement, while effects of challenging demands on work engagement are strengthened. These results are also important for other domains that are characterized by a high amount of teamwork.

Furthermore, the implications for practice relate to organizations, leaders, and human resource professionals to provide opportunities for teams and their members to learn and work together more successfully. Leaders and organizations can provide employees with opportunities for sharing their knowledge, creating new knowledge and structures and reflecting on tasks and teamwork. Furthermore, leaders can determine what and how often employees discuss or reflect on and thus trigger, cognitive as well as emotional aspects in teamwork. Especially regarding dealing with emotions in the team, leaders can show their emotions clearly within the team to stimulate team members’ perceptions and reactions and specifically address emotions in team meetings to trigger joint reflections and discussions and stimulate emotion regulation strategies. Furthermore, more work experience leads to less TLBs and dealing with emotions in the team. Especially with experienced team members, leaders could increase the required awareness about the importance of TLBs and dealing with emotions by emphasizing this importance in stressing the positive effects of TLBs and dealing with emotions. Furthermore, incentives can be provided for especially more experienced team members to for instance increase sharing and reflecting with the other team members on their knowledge.

There is evidence for several antecedents for TLBs and dealing with emotions in the team such as creating a safe and trustful climate within a team (Leicher and Mulder, 2016). Research indicates that when team members feel safe and work in a trustful environment, they more likely engage in feedback and reflection processes (Edmondson and Lei, 2014). Leaders and organizations can foster a safe and trustful climate by interventions and communication, while team members can foster safe team climate themselves by asking for feedback and initiating feedback processes. Furthermore, leaders could foster team behaviors by their leadership behavior (Koeslag-Kreunen et al., 2018).

In addition, in the process of recruiting new employees in the organization openness and commitment to join TLBs and dealing with emotions could be used as selection criteria. This could help human resource professionals that strive for optimal and effective team composition. Moreover, human resource professionals and team leaders can foster successful onboarding processes of new team members by having an eye for and stimulating the openness and commitment to TLBs and dealing with emotions. This can foster new team members work engagement, as well as their exchange and reflection on their work and the processes in the team which can strengthen the team as a social unit.

Due to the influence of team demands at work on TLBs, dealing with emotions in the team and team members’ work engagement organizations have several possibilities to strengthen demands with positive effects such as cognitive demands by for example fostering decision-making within a team. Furthermore, an organization could decrease negative effects of demands at work for instance by reducing hindrance demands for example by lingering the amount of emotional labor or avoiding conflicts that lead to negative emotions within the team.

### 5.4. Conclusion

Our study provides insights into the actual behavior of teams in the domain of care and assistance to the elderly, youth, or physically and/or mentally disabled, both on cognitive and emotional aspects. Furthermore, insights are provided for the capability of team learning behaviors and dealing with emotions in the team to mediate the relationship between team demands at work and team members’ work engagement as an important precondition for team and individual performance. The findings highlight the relation between cognitive and emotional aspects in the behavior of teams and may encourage future researchers and practitioners to address cognitive, emotional and motivational aspects in team research to provide a better understanding of team conditions, team behavior and team outcomes.

## Data availability statement

The data presented in this article are available only upon request due to privacy restrictions. Request to access should be directed to the corresponding author.

## Ethics statement

The studies involving human participants were reviewed and approved by the ethics committee of the university of Regensburg. The patients/participants provided their written informed consent to participate in this study.

## Author contributions

SG and RM developed the concept and study design, and wrote the manuscript. SG collected the data that was analyzed by SG and RM. All authors contributed to the article and approved the submitted version.

## Funding

The article processing charge was funded by the University of Regensburg in the funding programme Open Access Publishing.

## Conflict of interest

The authors declare that the research was conducted in the absence of any commercial or financial relationships that could be construed as a potential conflict of interest.

## Publisher’s note

All claims expressed in this article are solely those of the authors and do not necessarily represent those of their affiliated organizations, or those of the publisher, the editors and the reviewers. Any product that may be evaluated in this article, or claim that may be made by its manufacturer, is not guaranteed or endorsed by the publisher.
